# Multicenter phase II trial of preoperative chemoradiotherapy with S-1 for locally advanced oral squamous cell carcinoma

**DOI:** 10.1007/s00280-013-2101-5

**Published:** 2013-02-03

**Authors:** Hiroyuki Harada, Ken Omura, Hirofumi Tomioka, Hideki Nakayama, Akimitsu Hiraki, Masanori Shinohara, Yasuto Yoshihama, Satoru Shintani

**Affiliations:** 1 Department of Oral Surgery, Oral Restitution, Division of Oral Health Sciences, Graduate School, Tokyo Medical and Dental University, 1-5-45 Yushima, Bunkyo-ku, Tokyo, 113-8549 Japan; 2 Department of Oral and Maxillofacial Surgery, Faculty of Life Sciences, Kumamoto University, Kumamoto, Japan; 3 Department of Oral and Maxillofacial Surgery, School of Dentistry, Showa University, Tokyo, Japan

**Keywords:** Phase II trial, S-1, Oral squamous cell carcinoma, Preoperative chemoradiotherapy

## Abstract

**Purpose:**

We evaluated whether preoperative chemotherapy with S-1 and concurrent radiotherapy is feasible and efficacious in the treatment of advanced oral squamous cell carcinoma.

**Methods:**

Participants comprised 39 patients with oral carcinoma (stage III, *n* = 15; stage IVA, *n* = 24). All patients received a total radiation dose of 40 Gy, in once-daily 2-Gy fractions, and received S-1 at 65 mg/m^2^/day for 5 consecutive days, over 4 consecutive weeks with concurrent radiotherapy.

**Results:**

Hematological toxicity was mild and reversible. The most common non-hematological toxicity was grade 3 mucositis, but this was transient and tolerable. Radical surgery was performed for 37 patients, with the remaining 2 patients declining the surgery. Postoperatively, local failure developed in 1 patient, and neck failure in 2 patients. Distant metastases were identified in 4 patients. At a median follow-up of 38.0 months (range 23–88 months), locoregional control, disease-specific survival, and overall survival rates at 3 years were 91.5, 83.8, and 83.8 %, respectively.

**Conclusion:**

Concurrent administration of S-1 and radiotherapy combined with surgery offers a well-tolerated method of successfully treating advanced oral squamous cell carcinoma. The locoregional control rate remains high even at 3 years of follow-up, and no serious adverse effects have been encountered.

## Introduction

Achieving complete locoregional control is of utmost importance for head and neck squamous cell carcinoma, because distant metastases are seldom found at the first presentation. Although concurrent chemoradiotherapy has become standard in the treatment of locoregionally advanced squamous cell carcinoma of the head and neck [1–3], no consensus has been reached regarding the optimal combination and timing.

Radical ablative surgery followed by radio- or radiochemotherapy is frequently used for the treatment of advanced operable oral squamous cell carcinoma (OSCC). Although this combined therapeutic approach using a platinum-based chemotherapy protocol significantly enhances local tumor control [3], gross surgical resection is required to obtain clear surgical margins during the primary operation, frequently resulting in postoperative loss of organ function.

Preoperative chemoradiotherapy followed by surgery has become established in the clinical management of locally advanced operable OSCC over the last 20 years [4–9]. Most studies in the literature have used 40 or 50 Gy for preoperative radiotherapy, and cisplatin is often used as the radiosensitizing agent [4, 5, 7]. In a large study by Klug et al. [8] that summarized the results of preoperative chemoradiotherapy for oral and oropharyngeal cancer, the 5-year survival rate determined by meta-analysis of 32 studies (1,927 patients) was a remarkably good 62.6 %. Kirita et al. [9] reported obtaining a clinical response rate of 92.8 %, and a 5-year overall actuarial survival rate of 79.3 %, by treating advanced OSCC with preoperative cisplatin-based intravenous chemotherapy and concurrent radiotherapy at a total dose of 40 Gy. They concluded that their concurrent chemotherapy regimen was effective as a preoperative modality, with a relatively high response rate and acceptable level of adverse events.

S-1 is an oral fluoropyrimidine preparation that consists of tegafur, 5-chloro-2,4-dihydroxypyridine (gimeracil), a dihydropyrimidine dehydrogenase (DPD) inhibitor, and potassium oxonate (oteracil), which inhibits orotate phosphoribosyl transferase in the gastrointestinal tract, thereby reducing the gastrointestinal toxicity of 5-fluorouracil [10]. Preclinical studies using human oral cancer xenograft models have shown better responses from the combination of S-1 and fractionated radiotherapy than from either treatment alone [11].

We have already described the feasibility and efficacy of S-1 chemotherapy performed concomitantly with radiotherapy at a dose of 40 Gy of the preoperative treatment for advanced OSCC in a phase I trial [12]. The recommended dose of S-1 is 65 mg/m^2^/day for 5 days per week for 4 weeks with concurrent radiotherapy. The present study was designed as a phase II multicentric trial of preoperative chemotherapy with S-1 and concurrent radiotherapy for advanced OSCC. The primary end-point of this phase II study was the antitumor effect. The secondary end-points were clinical toxicities and overall survival.

## Materials and methods

### Patient eligibility

Previously untreated patients with histopathologically confirmed OSCC of stage III or IVA were evaluated for this study. Eligible patients were required to be from 20 to 75 years old, have an Eastern Cooperative Oncology Group performance status of 0 or 1, life expectancy ≥3 months, and adequate organ function (leukocytes 4,000/mm^3^, platelets ≥100,000/mm^3^, hemoglobin ≥9.0 g/dl, aspartate aminotransferase (AST) ≤2 times the upper normal limit (UNL), alanine aminotransferase (ALT) ≤2 times the UNL, alkaline phosphatase (ALP) ≤2 times the UNL, serum bilirubin ≤1.5 mg/dl, and serum creatinine ≤UNL).

Patients were excluded if they had received any prior systemic chemotherapy or radiotherapy, had a concomitant malignancy, active inflammatory bowel disease, active gastric/duodenal ulcer, active infection, severe heart disease, mental disorder, or other severe concurrent disease. Pregnant or lactating women were also excluded.

All study protocols were approved by the institutional review board at each participant center. All patients provided written informed consent before entry into this study.

### Treatment

We provided a fractional daily dose of 2 Gy 5 days/week, to a total dose of 40 Gy to the primary tumor site, and to the cervical nodes if the patient had nodal disease.

S-1 (Taiho Pharmaceutical, Tokyo, Japan) was administered orally twice a day after meals, concomitant with radiotherapy. Each capsule of S-1 contained either 20 or 25 mg of tegafur, and individual doses, calculated according to body surface area (BSA), were rounded down to the nearest pill size. S-1 dosing was as follows: BSA <1.25 m^2^, 50 mg/day; BSA 1.25–1.5 m^2^, 80 mg/day; BSA ≥1.5 m^2^, 100 mg/day, 5 days/week for 4 weeks with concurrent radiotherapy [12].

Adverse events were evaluated according to the National Cancer Institute Common Toxicity Criteria, version 3.0.

All patients had conventional surgical margins tattooed around the tumor. After completion of preoperative treatment, radical surgery was performed with resection of the primary tumor and/or neck dissection according to pretherapeutic staging data. The original extent of the tumor was resected with reference to the line of tattoo demarcation. Surgical reconstruction was undertaken using a range of locoregional flaps or microsurgical free flaps. Neck dissection was required for cases with the presence of clinically palpable cervical lymph node metastasis or transfer of microsurgical flaps.

### Treatment evaluation

Radical surgery was performed for 37 of the 39 patients, with the other 2 patients declining to undergo the surgery. We judged the clinical efficacy of the chemoradiotherapeutic protocol immediately before surgery. The median interval between end of chemoradiotherapy and surgery was 22.5 days (range 13–36 days).

Evaluation methods included computed tomography (CT), magnetic resonance imaging (MRI), and ultrasonography. Responses at the primary site and neck were analyzed separately. Treatment effects were estimated according to the RECIST^1.0^.

For the histological evaluation of primary tumors, we applied the classification of therapeutic effectiveness described by Shimosato et al. [13]: grade 0, no noticeable change; grade I, minimal cellular changes, but the majority of tumor cells appear viable; grade IIa, despite the presence of cellular changes and partial destruction of the tumors, the tumor is still readily recognizable, and many tumor cells appear viable; grade IIb, tumor destruction is extensive, but viable cell nests are present in small areas of tumor (one-quarter of tumor mass, excluding areas of coagulative necrosis); grade III, only a few scattered, markedly altered, and presumably non-viable tumor cells present, singly or in small clusters, and few or no viable cells are seen; and grade IV, no tumor cells remaining in any section.

### Statistical analysis

Survival was assessed from the first day of treatment until death or last patient contact. Overall and cumulative survival rates were calculated according to the Kaplan–Meier method [14].

## Results

### Patient characteristics

Participants comprised 39 patients (24 men, 15 women) enrolled in this study between March 2005 and August 2010. All patients received the preoperative chemoradiotherapy with S-1, as planned. Of the 39 patients, 37 (94.9 %) underwent radical surgery. The remaining 2 patients declined the surgery, and received brachytherapy and a total of 70 Gy radiotherapy each. Median age was 56.5 years (range 21–75 years) and Eastern Cooperative Oncology Group (ECOG) score was 0 for 38 patients and 1 for 1 patient. Primary lesion sites were the tongue (*n* = 20), floor of the mouth (*n* = 2), maxillary gingiva (*n* = 4), mandibular gingiva (*n* = 12), and hard palate (*n* = 1). TN classifications are shown in Table 1. Fifteen patients had stage III carcinoma and 24 had stage IV. Median duration of follow-up was 38.0 months (range 23–88 months).

**Table 1:** TN classification

	T2	T3	T4a	Total
N0	0	6	7	13
N1	7	2	2	11
N2b	3	4	5	12
N2c	0	1	2	3
Total	10	13	16	39

Reconstruction was performed using microvascular transfer in 30 patients, split-thickness skin graft in 3 patients, and primary closure in 4 patients. A total of 3 patients had no neck dissection, 30 patients underwent unilateral neck dissection, and 4 patients had bilateral neck dissection.

### Toxicity

Cases with toxicities observed during treatment or within 2 weeks after chemoradiotherapy are listed in Table 2. Grade 1–2 leukocytopenia was observed in 17 patients (43.6 %). Neutropenia was rare; grade 1–2 neutropenia occurred in 6 patients (15.4 %). Grade 1–2 anemia was observed in 11 patients (28.2 %). In this study, all hematological toxicities were mild and reversible, and no grade 3 or 4 hematological toxicity was encountered.

**Table 2:** Prevalence of adverse events

Toxicity^a^	Grade		
	1	2	3
Hematological toxicity			
Leukocytopenia	8	9	0
Neutropenia	3	3	0
Hemoglobin	8	3	0
Thrombocytopenia	1	0	0
AST	4	1	0
ALT	3	2	0
Non-hematological toxicity			
Anorexia	5	3	0
Fatigue	1	0	0
Dermatitis	24	8	0
Mucositis	1	5	33

*AST* aspartate aminotransferase, *ALT* alanine aminotransferase

^a^Toxicities were defined according to the National Cancer Institute Common Toxicity Criteria, version 3.0

Non-hematological toxicities, apart from mucositis, were grade 1 or 2, and the most common was mucositis. All patients experienced grade 1–3 mucositis, and 33 patients (84.6 %) had grade 3 mucositis. However, mucositis was transient and tolerable in all cases. Treatment was not interrupted due to adverse events in any cases.

### Efficacy

Clinical responses of primary tumors are shown in Table 3. Five patients achieved complete response (CR) and 27 achieved partial response (PR). The clinical response rate (CR + PR) was 100 % in T2, 84.6 % in T3 and 68.8 % in T4a. Overall clinical response rate (CR + PR) was 82.1 %. The histological evaluation was grade IV (no viable tumor cells in any section) in 7 patients (Table 4) and grade III in 8. Histological response rate, defined as grade IIb, III, or IV, was 78.4 %.

**Table 3:** Clinical response of primary tumors

	CR	PR	SD	PD	Response rate (%)
T2	4	6			100
T3		11	2		84.6
T4a	1	10	4	1	68.8
Total	5	27	6	1	82.1

*CR* complete response, *PR* partial response, *SD* stable disease, *PD* progressive disease

**Table 4:** Histological evaluations of primary tumors after chemoradiotherapy

Grade^a^	IV	III	IIb	IIa	I	Response rate
No. of cases	7	8	14	6	2	78.4 %

^a^Histological evaluations were defined according to the classification of therapeutic effectiveness described by Shimosato et al. [13]

Clinical responses of neck metastases are shown in Table 5. Thirteen patients showed clinical PR, 10 showed SD, and 3 showed PD. Clinical response rate (CR + PR) for neck disease was 50.0 %.

**Table 5:** Clinical response of neck disease

Clinical response	CR	PR	SD	PD	Response rate
No. of cases	0	13	10	3	50.0 %

*CR* complete response, *PR* partial response, *SD* stable disease, *PD* progressive disease

Postoperative chemotherapy was performed in three patients that had cervical lymph nodes metastasis more than four. The chemotherapy was administered 2 cycles with a 4-week interval, with a regimen of cisplatin 80 mg/m^2^ (day 1) and 5-fluorouracil 800 mg/m^2^/day (day 1–5).

**Fig. 1:**
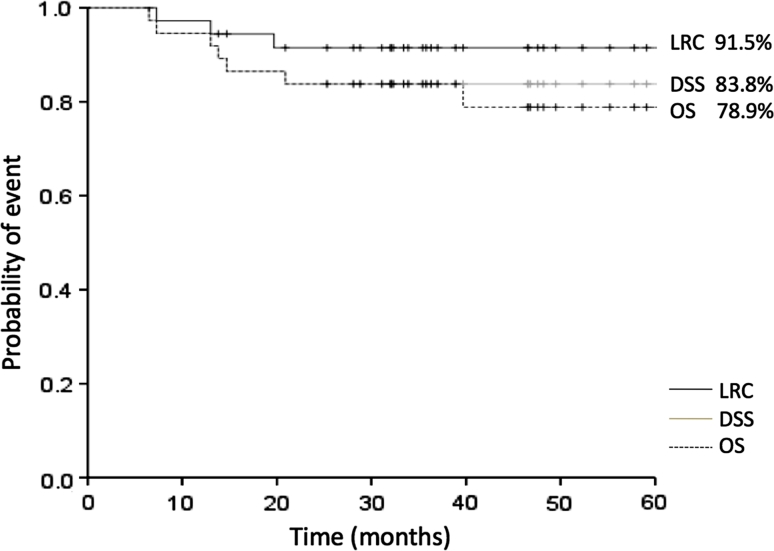
Locoregional control rate, disease-specific survival, and overall survival for 37 patients treated with preoperative chemoradiotherapy followed by surgery

After surgery, local failure developed in 1 patient, and neck failure in 2 patients. Distant metastases were identified in 4 patients. With a median follow-up of 38.0 months (range 23–88 months), locoregional control rate (LRC), disease-specific survival rate (DSS) and overall survival rate (OS) at 3 years were 91.5, 83.8 and 83.8 %, respectively (Fig. 1). Five-year LRC, DSS and OS were 91.5, 83.8 and 78.9 %, respectively.

## Discussion

We set out to determine the feasibility and efficacy of S-1 with concurrent radiotherapy for advanced OSCC. The regimen of preoperative chemoradiotherapy was tolerated well, and patients assigned this treatment showed no disadvantages at the time of surgical treatment.

Tsukuda et al. [15] reported that most adverse events associated with S-1 administration alone were hematological, gastrointestinal, or skin toxicities, and most were grade 1 or 2 and controllable. In the present study, no severe hematological, gastrointestinal, or skin toxicities were encountered. Mucositis was the most common adverse event, with grade 3 mucositis observed in 84.6 % of patients. However, treatment was not interrupted by adverse events in any patient. This treatment is possible to administer on an outpatient basis.

In this study, overall clinical response rate (CR + PR) of the primary tumor was 82.1 %, and the histological response rate (grade IIb, III, or IV) was 78.4 %. Most studies in the literature have used 40 or 50 Gy for preoperative radiotherapy, and cisplatin is often used as a radiosensitizing agent [4, 5]. In several reports concerning preoperative chemoradiotherapy, 5-year OS has ranged from 62.6 to 79.3 % [4, 5, 7, 8, 16, 17]. In the present study, LRC, DSS and OS at 3 years were 91.5, 83.8 and 83.8 %, respectively. In addition, fewer toxic effects were seen with the present study than with previous investigations.

On the other hand, the clinical response rate for neck nodal disease was 50.0 %. This result was poor compared with the clinical response rate of the primary tumor. A late phase II clinical study of S-1 alone found a clinical response rate of 21.7 % for cervical lymph node metastasis [18]. These results suggest that neck dissection is warranted for metastatic lymph nodes in patients with oral carcinoma.

The theoretical advantages of preoperative chemoradiotherapy are downstaging of the primary tumor, increased resectability rate, and the elimination of micrometastasis. The preoperative chemoradiotherapy protocol with a radiation dose of 40 Gy might offer several therapeutic and prognostic advantages. Limiting the dose to 40 Gy before surgery reduced the overall radiation dose for the patient and the organ-specific dose for salivary glands, facilitating the preservation of salivary gland function and resulting in less post-therapeutic xerostomia [19, 20]. Osteoradionecrosis of the jaw is one of the serious complications of radiotherapy for head and neck cancer. High-dose radiotherapy for oral cancer induces mandibular osteoradionecrosis with an incidence of approximately 5–20 % [21, 22]. This procedure with 40 Gy appears likely to reduce the incidence of osteoradionecrosis.

Furthermore, the risk of wound-healing disorders, which result from decreased vascularization of the pre-irritated recipient tissue after reconstructive surgery with free-flap transfers, is significantly reduced with a radiation dose of 40 Gy compared to radiation doses >60 Gy [23].

Another advantage of neoadjuvant therapy followed by surgery might be adequate histopathological diagnosis of the residual tumor, which is superior to clinical and radiological assessment. Analysis of the antitumor effect and margin study of the primary tumor is crucial. The pattern of cervical lymph node metastasis is also important. These findings are useful for predicting prognosis and the necessity for adjuvant treatment.

In conclusion, concurrent administration of S-1 and radiotherapy combined with surgery offers a well-tolerated and successful method for treating advanced oral cancer. LRC remains high even after 3 years of follow-up, and serious adverse effects appear to be reduced. The effectiveness of different neoadjuvant protocols presented in the literature should be evaluated in prospective randomized studies in the future.
